# Enhanced visualization in endoleak detection through iterative and AI-noise optimized spectral reconstructions

**DOI:** 10.1038/s41598-024-54502-1

**Published:** 2024-02-15

**Authors:** Wojciech Kazimierczak, Natalia Kazimierczak, Justyna Wilamowska, Olaf Wojtowicz, Ewa Nowak, Zbigniew Serafin

**Affiliations:** 1 Collegium Medicum, Nicolaus Copernicus University in Torun, Jagiellońska 13-15, 85-067 Bydgoszcz, Poland; 2 Kazimierczak Private Medical Practice, Dworcowa 13/u6a, 85-009 Bydgoszcz, Poland; 3 University Hospital No 1 in Bydgoszcz, Marii Skłodowskiej – Curie 9, 85-094 Bydgoszcz, Poland

**Keywords:** Abdominal aortic aneurysms, Endoleak, Endovascular aneurysm repair, Dual-energy computed tomography angiography, Virtual monoenergetic images, Image reconstruction, deep learning model, Adaptive statistical iterative reconstruction, Aneurysm, Aortic diseases, Diagnosis, Computed tomography

## Abstract

To assess the image quality parameters of dual-energy computed tomography angiography (DECTA) 40-, and 60 keV virtual monoenergetic images (VMIs) combined with deep learning-based image reconstruction model (DLM) and iterative reconstructions (IR). CT scans of 28 post EVAR patients were enrolled. The 60 s delayed phase of DECTA was evaluated. Objective [noise, contrast-to-noise ratio (CNR), signal-to-noise ratio (SNR)] and subjective (overall image quality and endoleak conspicuity – 3 blinded readers assessment) image quality analyses were performed. The following reconstructions were evaluated: VMI 40, 60 keV VMI; IR VMI 40, 60 keV; DLM VMI 40, 60 keV. The noise level of the DLM VMI images was approximately 50% lower than that of VMI reconstruction. The highest CNR and SNR values were measured in VMI DLM images. The mean CNR in endoleak in 40 keV was accounted for as 1.83 ± 1.2; 2.07 ± 2.02; 3.6 ± 3.26 in VMI, VMI IR, and VMI DLM, respectively. The DLM algorithm significantly reduced noise and increased lesion conspicuity, resulting in higher objective and subjective image quality compared to other reconstruction techniques. The application of DLM algorithms to low-energy VMIs significantly enhances the diagnostic value of DECTA in evaluating endoleaks. DLM reconstructions surpass traditional VMIs and IR in terms of image quality.

## Introduction

Abdominal aortic aneurysms (AAA) are a common medical condition that affects approximately 5% of the general population^1^. Abdominal aortic aneurysms are also one of the leading causes of death in developed countries and are estimated to be one in ten causes of death^1^. Currently, the most commonly available and preferred treatment for AAA is endovascular aortic repair (EVAR)^2^. Advances in endovascular procedures and materials have enabled the use of branched and fenestrated f/bEVAR stentgrafts and the extension of EVAR recommendations to perirenal and thoracoabdominal aneurysms. However, the greater complexity of procedures and devices is associated with a higher incidence of complications, especially endoleaks^3^. Endoleak is an unique to this population and potentially life-threatening complication defined as the persistent leakage of blood, beyond the stentgraft lumen into the aneurysmal sac^4^. Due to the possible further sac expansion and subsequent rupture, EVAR patients require lifelong diagnostic follow-up^2,5^.

Computed tomography angiography (CTA) is the basic diagnostic modality for the follow-up of patients after endovascular aneurysm repair (EVAR)^6^. Dual-energy CTA (DECTA), a modification of CTA, has already proven its high diagnostic value in various vascular conditions as well as in post-EVAR imaging^7,8^. According to the available literature, DECTA has a high diagnostic value, exceeding that of standard single energy CT (SECT) in numerous vascular applications, including the detection of pulmonary embolism, assessment of coronary arteries, functional assessment of the myocardium, differentiation of venous thrombosis from iodine flow artifacts, detection of active arterial bleeding, and reduction of contrast agent and radiation doses^9–14^.

The primary advantage of DECT is its ability to reconstruct virtual monoenergetic images (VMIs), which mimic the attenuation values of an image acquired at a single energy level (typically within the range of 40–200 keV). In DECTA, vessel contrast is enhanced as the energy level of the monochromatic images decreases, similar to standard SECT performed at a low tube voltage, such as 80 or 100 kVp. Research has shown that low-keV images can better reveal subtle contrast enhancements compared to single-energy CT polychromatic images due to higher beam attenuation from iodine^15^. Generally, low-keV images (40–70 keV) are advantageous for increasing iodine contrast but also lead to higher noise levels^16^. Low-keV datasets have already proven their high diagnostic value in endoleak detection^17–22^. However, such images are associated with higher noise levels and artifacts^16^. Noise manifests as inconsistent attenuation values in projection images, causing errors in the computed attenuation coefficient and reducing the low-contrast resolution, which affects the differentiation of low-density tissues^23,24^. Both artifacts and noise may simulate or obscure pathologies, leading to misdiagnosis and potentially worsening patient outcomes.

To address the challenge of image noise and improve image quality parameters, iterative reconstructions (IR), such as adaptive statistical iterative reconstruction (ASIR, GE Healthcare, Milwaukee, WI, USA), were introduced. ASIR creates mixed images between filtered back projection (FBP) and IR, with IR percentages ranging from 0 to 100%, where the percentage indicates the contribution of ASIR to the final images. The most common application of IR is to optimize image quality while reducing the radiation dose while maintaining image quality parameters. Such reconstructions allow for the reduction of image noise and artifacts as well as the improvement of diagnostic confidence and increased conspicuity of subtle abdominal lesions and cardiovascular conditions^25–28^. IR has already proven its potential in maintaining image quality parameters while enabling significant radiation dose reduction^29,30^.

Recently, deep-learning-based image reconstruction algorithms (DLRs) have emerged as an alternative technical approach. DLRs have already been proven to improve diagnostic accuracy, while decreasing noise and reducing radiation doses^31–34^. However, their use may be restricted to a single software and CT scanner vendor (e.g., AiCE by Canon Medical Systems and TrueFidelity™ by GE Healthcare), preventing their use with scans acquired from other manufacturers. The solution presented is a vendor-agnostic deep learning model (DLM) that operates in the image post-processing domain and does not require projection data. ClariCT.AI (ClariPI, Seoul, Korea), a specific example of such a DLM solution, has effectively shown that DLMs can both minimize image noise and achieve a high diagnostic accuracy comparable to that of vendor-specific DLRs^35–38^.

ClariCT.AI obtained FDA clearance in 2019 and trained their algorithm using noise-simulated FBP images as input and routine-dose FBP images as ground truth. ClariCT.AI is an example of indirect, image based DLR framework—the neural network optimizes the image after initial reconstruction with FBP or IR^39^. To develop the algorithm, the training data consisted of over a million CT images from four CT vendors and various reconstruction parameters^40^. In a study by Nam et al.^41^, ClariCT.AI was compared to HIR on 100 lower-dose follow-up chest CT scans, with ClariCT.AI demonstrating superior noise appearance, spatial resolution, and overall image quality. Similar results were achieved by Park et al.^42^, where the authors showed non-inferior image quality of DLM reconstructed images with 67% radiation dose reduction, compared to standard radiation dose MBIR reconstructions. Therefore, it is particularly interesting to compare the potential of both approaches for noise reduction and assess their capabilities in vascular imaging with images marked by high levels of noise, such as in the case of low-energy-level VMIs. To the best of our knowledge, no study has analyzed the impact of both solutions on imaging endoleaks in patients after EVAR procedures.

The aim of the study was to evaluate low-energy VMI reconstruction techniques with and without IR and DLM reconstructions in the assessment of endoleaks after f/brEVAR procedures.

## Results

### Patient population

A total of 28 patients (5 women, 23 men; mean age 72.1 years, range 62.9–86.9) were included in the study. All CT scans were performed 30 days after the stent graft implantation. During the study, 28 sets of 6 reconstructions were quantitatively and qualitatively evaluated.

The sample size was determined using an online sample size calculator (https://clincalc.com; accessed on November 22, 2023). To assess the adequacy of our study group size, we evaluated the minimal statistically significant differences in the mean CNR values for VMI at 40 keV and 60 keV. The calculated minimal significant differences were 0.86 for a VMI 40 keV and 0.83 for VMI 60 keV. These values were significantly lower than the differences observed between the CNR for VMI 40 keV and DLM VMI 40 keV (1.53), and between VMI 60 keV and DLM VMI 60 keV (1.58).

### Objective image quality

**Figure 1:**
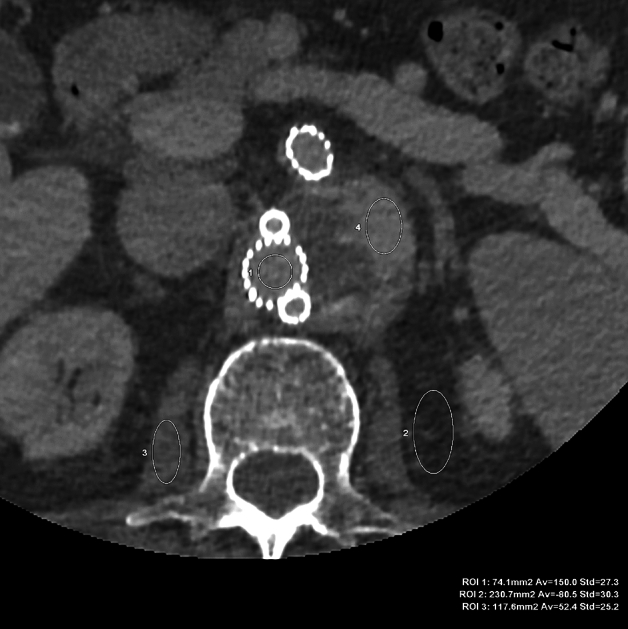
VMI 60 keV axial image for objective image quality evaluation. ROIs placed in the main stentgraft module (ROI 1 – 150 HU, SD 27.3), adipose tissue (ROI 2 – Av. − 80.5 HU, SD 30.3), psoas muscle (ROI 3 – Av. 52.4 HU, SD 25.2), endoleak (ROI 4 – Av. 145.2 HU, SD 27.3). The same window settings (W 800, L 100), patient one month after EVAR.

The results of the objective image quality assessment of all the evaluated reconstructions are summarized in Table 1. Figure 1 shows an example of ROI positioning.

**Table 1:** Objective image quality parameters of the aorta and endoleaks.

Parameter		VMI 40 keV (A)	VMI 60 keV (B)	IR 60% VMI 40 keV (C)	IR 60% VMI 60 keV (D)	DLM VMI 40 keV (E)	DLM VMI 60 keV (F)	*p*
CT attenuation, [HU]	Aorta	390.12 ± 182.82	193.43 ± 85.43	368.72 ± 125.66	180.99 ± 59.75	388.31 ± 180.55	192.52 ± 85.27	*p* < 0.001; A, E, C > B, F, D B > F
	Endoleak	238.95 ± 153.48	125.24 ± 69.29	226.88 ± 89.81	116.86 ± 40.65	236.74 ± 154.56	123.42 ± 70.11	*p* < 0.001; A, E, C > B, F, D
Noise		83.96 ± 23.56	41.02 ± 10.61	79.78 ± 18.4	39.79 ± 9.79	45.07 ± 12.98	20.79 ± 6.44	*p* < 0.001; A, C > E, B > D > F
CNR	Aorta	3.89 ± 2.57	3.4 ± 2.42	4.2 ± 3.04	3.78 ± 2.92	7.74 ± 6.06	7.7 ± 6.68	*p* < 0.001; E, F > C, A, D, B A > B C > D
	Endoleak	1.83 ± 1.2	1.61 ± 0.91	2.07 ± 2.02	1.88 ± 1.78	3.6 ± 3.26	3.46 ± 2.91	*p* = 0.001; E, F > C, D, A, B
SNR	Aorta	4.98 ± 2.87	4.95 ± 2.82	5.36 ± 3.18	5.37 ± 3.16	9.87 ± 6.57	10.89 ± 7.65	*p* < 0.001; F,E > D, C, B, A
	Endoleak	2.79 ± 1.37	2.9 ± 1.27	3.06 ± 2.07	3.2 ± 1.92	5.43 ± 3.44	6.01 ± 3.5	*p* < 0.001; F, E > D, C, B, A

CT attenuation and CNR, SNR given as means ± standard deviation.

VMI, virtual monoenergetic images; IR, iterative reconstructions; DLM, deep learning model; CNR, contrast-to-noise ratio; HU, hounsfield units; SNR, signal-to-noise ratio.

**Figure 2:**
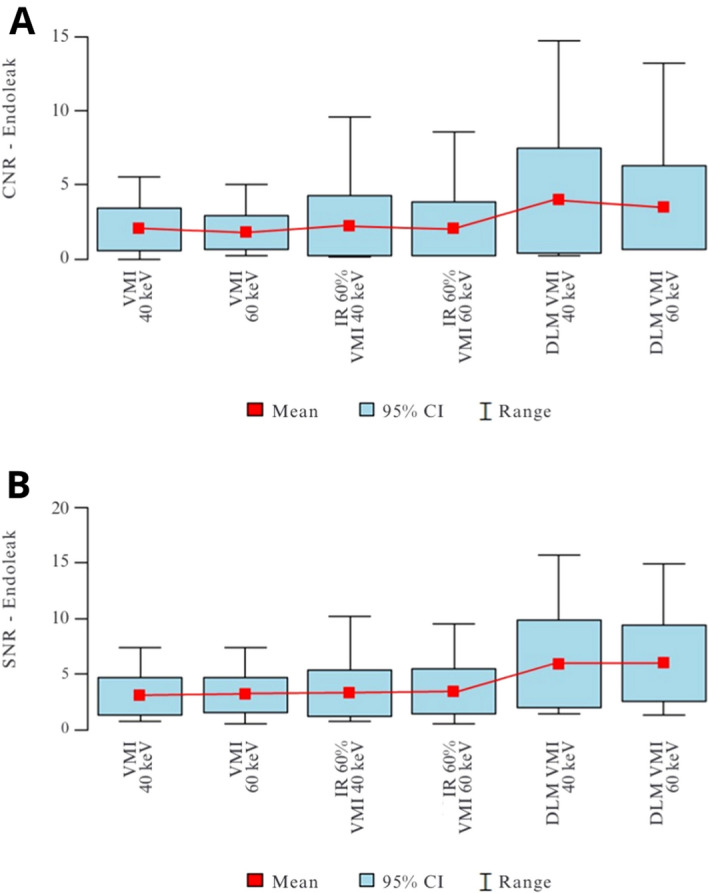
(**A**,**B**) CNR (**A**) and SNR (**B**) in the endoleaks in VMI, VMI IR, and VMI DLM reconstructions.

There are significant differences between the 40- and 60-keV VMI images in all three sets of reconstructions. Differences in noise levels between the VMI and VMI IR reconstruction were not statistically significant. The noise level of the VMI DLM images was roughly 2 × lower than that for VMI reconstructions (VMI 40 keV = 83.96 ± 23.56; DLM 40 keV VMI = 45.07 ± 12.98). However, slight but statistically significant differences between the SNR and CNR values between VMI and VMI IR 60% were observed. The highest CNR and SNR values were measured for VMI DLM images. The mean CNR in endoleaks in 40 keV was accounted for as 1.83 ± 1.2; 2.07 ± 2.02; 3.6 ± 3.26 in VMI, VMI IR, and VMI DLM, respectively. Figure 2 shows the results of the CNR and SNR calculations for the endoleaks.

**Figure 3:**
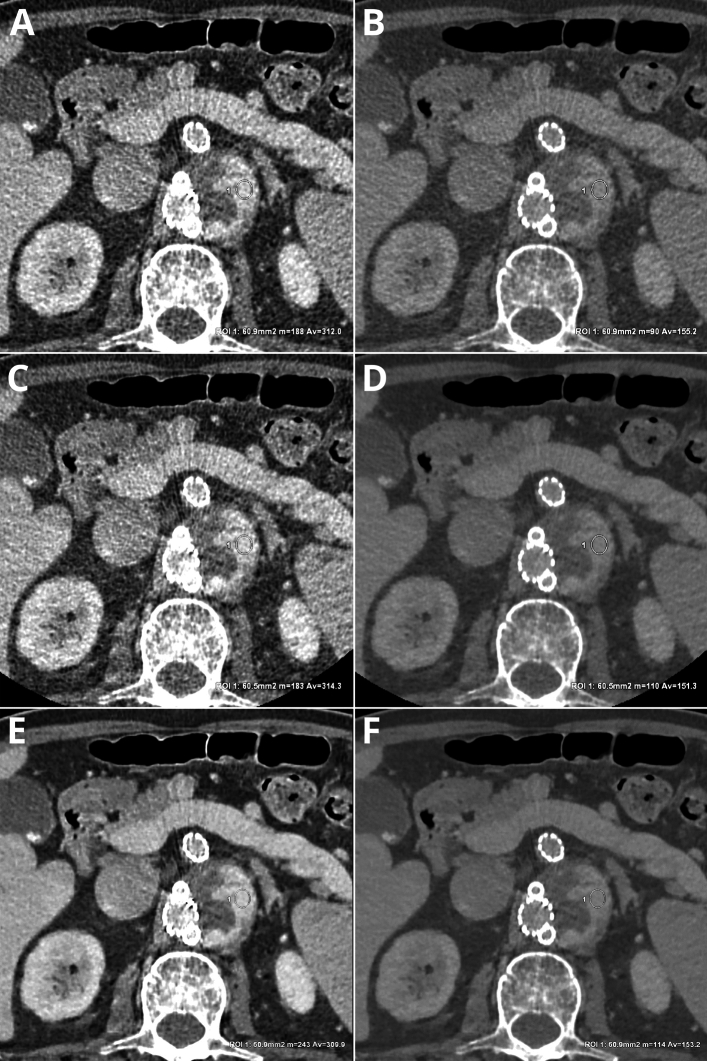
Sample ROI measurements in type III endoleak in: VMI 40 keV – Av. 312.0 HU, SD 94.9 (**A**), VMI 60 keV – Av. 155.2 HU, SD 50.6 (**B**), IR 60% VMI 40 keV – Av. 314.3 HU, SD 85.6 (**C**), IR 60% VMI 60 keV – Av. 151.3 HU, SD 42.0 (**D**), DLM VMI 40 keV – Av. 309.9 HU, SD 76.9 (**E**), DLM VMI 60 keV – Av. 153.2 HU, SD 39.7 (**F**). The same window settings (W 800, L 100), patient one month after EVAR.

An example of endoleak ROI measurements in all assessed reconstructions is shown in Fig. 3.

### Subjective image quality

**Figure 4:**
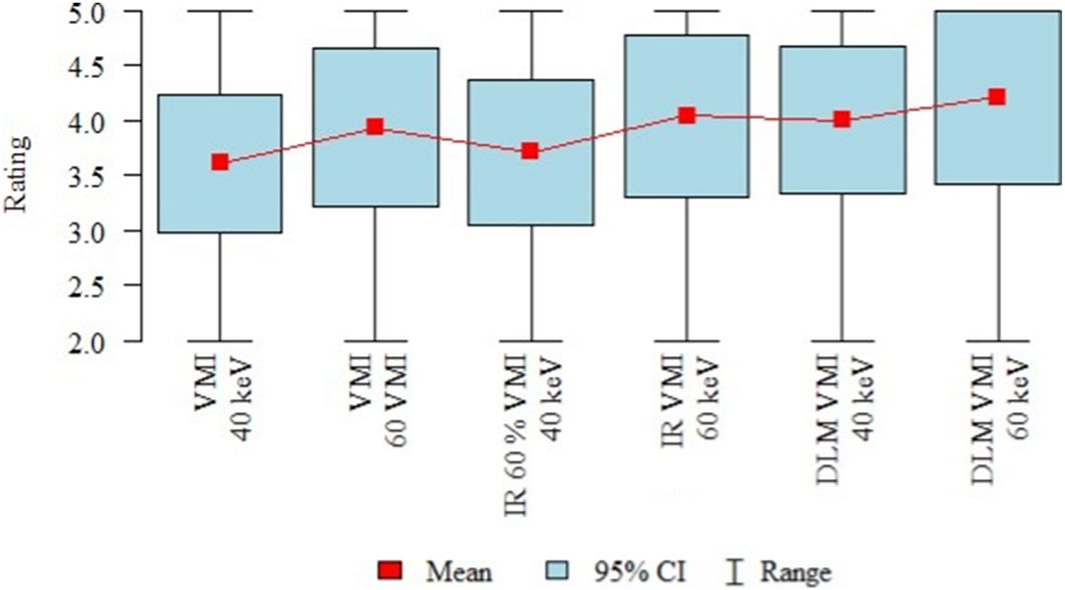
Results of the overall image quality assessment (mean values, 95% confidence intervals (CI), ranges).

The results of the subjective image quality assessments performed by the three readers are summarized in Table 2 and Fig. 4.

**Table 2:** Overall subjective image quality (3 readers).

Rating	VMI 40 keV (A)	VMI 60 VMI (B)	IR 60% VMI 40 keV (C)	IR VMI 60 keV (D)	DLM VMI 40 keV (E)	DLM VMI 60 keV (F)	*p*
Mean ± SD	3.61 ± 0.63	3.93 ± 0.72	3.71 ± 0.66	4.04 ± 0.74	4 ± 0.67	4.21 ± 0.79	*p* < 0.001*
Median	4	4	4	4	4	4	F > C, A D > A
Quartiles	3–4	4–4	3–4	4–4.25	4–4	4–5	

p—Friedman test + post-hoc analysis (Wilcoxon paired tests with Bonferroni correction).

*Statistically significant (*p* < 0.05).

SD, standard deviation; VMI, virtual monoenergetic images; IR, iterative reconstructions; DLM, deep learning model.

The data in the tables represent the mean ratings of three readers. Overall, the subjective image quality was the lowest for VMI 40 keV (mean rating 3.61 ± 0.63), and this was significantly lower than that of DLM VMI 60 keV and IR 60% VMI 40 keV (*p* < 0.001). The highest subjective image quality was observed with DLM VMI 60 keV (mean 4.21 ± 0.79). The objective image quality analysis results showed significant differences between the 40- and 60-keV VMI images across all three sets of reconstructions.

**Figure 5:**
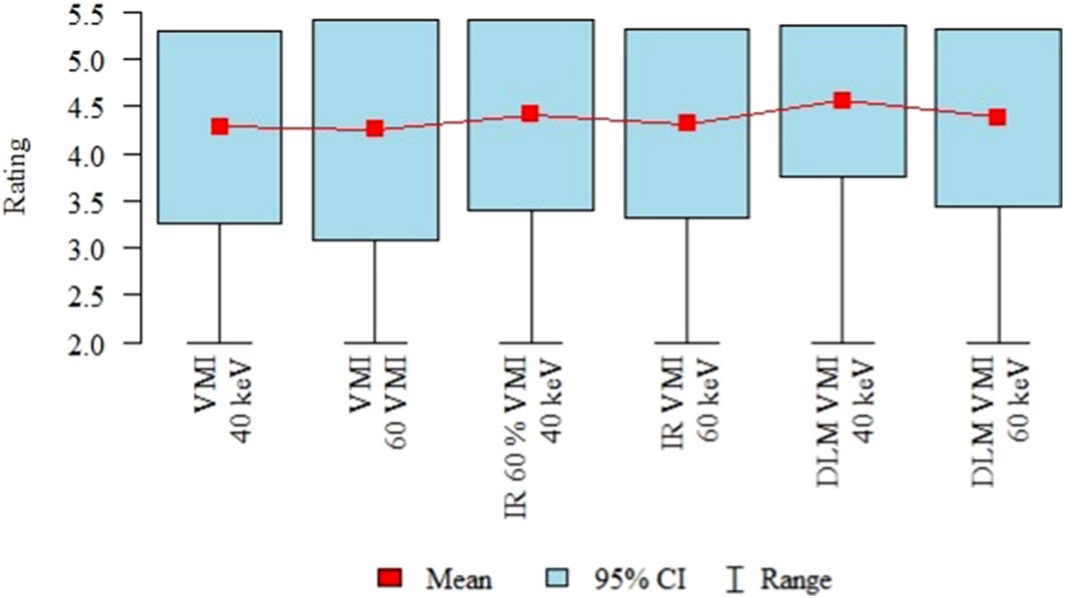
Results of the endoleak conspicuity assessment (mean values, 95% confidence intervals (CI), ranges).

The results of the subjective endoleak presence assessment are summarized in Table 3 and Fig. 5. The endoleak presence assessment using a 5-point CS showed the lowest ratings for VMI 60 keV (mean rating 4.25 ± 1.16) and the highest for DLM VMI 40 keV (mean rating 4.56 ± 0.80); the difference was statistically significant (*p* = 0.012). DLM VMI 40 keV ratings also showed the smallest variability (SD = 0.80). It is worth noting that the results of the endoleak presence certainty analysis revealed very high values across all analyzed reconstructions (4.25–4.56). The inter-reader concordance of the image quality and endoleak conspicuity assessments was fair (Table 4).

**Table 3:** Subjective endoleak presence confidence assessment (3readers).

Rating	VMI 40 keV (A)	VMI 60 VMI (B)	IR 60% VMI 40 keV (C)	IR VMI 60 keV (D)	DLM VMI 40 keV (E)	DLM VMI 60 keV (F)	*p*
Mean ± SD	4.28 ± 1.02	4.25 ± 1.16	4.41 ± 1.01	4.31 ± 1	4.56 ± 0.8	4.38 ± 0.94	*p* = 0.012*
Median	5	5	5	5	5	5	F > B
Quartiles	4–5	4–5	4–5	4–5	4–5	4–5	

p—Friedman test + post-hoc analysis (Wilcoxon paired tests with Bonferroni correction).

*Statistically significant (*p* < 0.05).

SD, standard deviation; VMI, virtual monoenergetic images; IR, iterative reconstructions; DLM, deep learning model.

**Table 4:** Subjective image quality inter-reader agreement assessment.

Parameter	ICC	95% CI		Agreement (Cicchetti)	Agreement (Koo & Li)
VMI 40 keV	0.538	0.337	0.717	Fair	Fair
VMI 60 keV	0.585	0.392	0.750	Fair	Fair
IR VMI 40 keV	0.556	0.356	0.729	Fair	Fair
IR VMI 60 keV	0.586	0.389	0.752	Fair	Fair
DLM VMI 40 keV	0.467	0.256	0.663	Fair	Poor
DLM VMI 60 keV	0.495	0.285	0.685	Fair	Poor

ICC, Interclass Correlation Coefficient; CI, Confidence Interval; VMI, Virtual Monoenergetic Images; IR, Iterative Reconstructions; DLM, Deep Learning Mode.

### Error study

Analysis of the repeatability of subjective image quality analysis carried out by the reader demonstrated excellent concordance (ICC = 0.837).

## Discussion

In this study, we aimed to compare the image quality parameters of different postprocessing algorithms for the detection of endoleaks in patients after EVAR. Our findings revealed that DLM-based noise reduction 40 and 60 VMI highly surpasses both subjective and objective image quality of conventional VMIs and IR VMIs. Our comparison between the VMI and IR VMI images revealed slight but statistically significant differences in CNR, SNR, and noise levels. The results of the subjective image quality assessment showed the highest endoleak conspicuity and overall image quality ratings for DLM reconstructions. The results of the qualitative analysis showed that the highest endoleak conspicuity rating was given to the DLM VMI 60 keV.

Considering the available literature and our own observations, we have made an initial selection of the analyzed VMIs at 40 and 60 keV. The choice of reconstruction presenting low-energy levels of VMIs was dictated by the higher absorption of low-energy photons by iodine. These properties result from the k-edge of iodine (33.2 keV) and constitute the main advantage of VMI angiographic imaging^43^. Previous studies have shown that 40 keV VMIs allow for the highest CT number values in angiographic studies; however, they are burdened with significant noise^44–46^. A stepwise decrease in image noise was observed with an increase in VMI energy levels; however, it was accompanied by a significant decrease in CT attenuation, with the lowest values at the highest photon energies^47^. Therefore, a series of studies indicate the usefulness of 60 keV reconstructions in angiographic studies, which on one hand show high contrast attenuation, and on the other hand, acceptable image noise levels, resulting in high CNR and SNR values^48–50^. Both chosen reconstructions have already proven high image quality parameters in angiographic studies, as well as in endoleak detection^17–22^. In a study by Maturen et al.^17^, 55 keV VMIs had higher endoleak conspicuity ratings compared to VMI 75 keV. Charalombous et al.^22^ reported a 54 keV VMI to enhance the endoleak detection efficiency. In a recent study in 2023, Kazimierczak et al.^19^ showed a high diagnostic value of 40-keV VMIs in endoleak detection, exceeding LB reconstructions. Our results clearly show that despite lower CNR levels, both subjective and overall image quality and endoleak conspicuity ratings were higher for 60 keV compared to 40 keV reconstructions.

The findings of this study highlight the potential advantages of utilizing low-energy VMI reconstruction combined with advanced reconstruction techniques such as IR and DLM to evaluate endoleaks after f/brEVAR procedures. The objective and subjective image quality parameters were significantly higher for IR and DLM reconstructions than for standard VMIs. As indicated before, low-energy VMIs have great potential for DECTA endoleak detection^17–21^, and noise-optimized reconstructions allow for either improved endoleak conspicuity^18^ or significant radiation dose reduction while maintaining image quality^51,52^. Martin et al.^18^ investigated the image quality parameters and diagnostic accuracy of VMI and noise-optimized VMI reconstructions for endoleak detection. Both the image quality parameters (CNR) and results of the ROC analysis for endoleak detection were significantly higher in VMI and noise-optimized VMI reconstructions. The literature also indicates numerous successful applications of IR algorithms in low-radiation dose protocols^53,54^. Studies by Hansen et al. and Naidu et al.^51,52^ showed a potential 72–73% radiation dose reduction with model-based iterative reconstruction (MBIR), with comparable image quality parameters and preserved diagnostic accuracy. However, despite the promising results, this technique is not widely used because of the prolonged reconstruction time and the artificial, “plastic” appearance of the images^39^. Therefore, it was particularly interesting to compare the image quality parameters of both 40 and 60 keV VMIs reconstructed using the two different noise reduction approaches, iterative and AI-based.

A recent breakthrough in AI technology, resulting in the development of generative AI, has led to the creation of AI-driven noise optimization tools that utilize DLR algorithms, surpassing the capabilities of iterative reconstruction (IR), including MBIR^39^. A few studies have analyzed the performance of the evaluated DLM tool (ClariCT.AI)^41,55,56^. Nam et al.^41^ compared the subjective image quality of ClariCT.AI with iterative reconstruction (IR) on 100 low-dose chest CT scans. The noise level, spatial resolution, and overall image quality of ClariCT.AI were superior to those of IR. In a recent study, Seo et al.^55^ proved that DLM VMI 40 keV images exhibited greater noise reduction, better lesion conspicuity, enhanced image contrast, and higher overall image quality than IR in patients with hypervascular liver lesions. In a similar study, Lee et al.^56^ demonstrated that DLM VMI 40 keV provided better image quality and comparable diagnostic performance in the detection of hypovascular hepatic lesions compared to VMI 40 keV.

Few studies have been conducted on the performance of DLM algorithms for cardiac and vascular CT^32,57–60^. In cardiac CT, vessel contouring is adequate with routine-dose FBP, IR, and MBIR, but deteriorates with low-dose scans due to increased noise and reduced spatial resolution. However, DLRs have the potential to overcome the limitations of these techniques^57,58^. In one of the first published studies in this area, Tatsugami et al.^39^ demonstrated that DLR reduced image noise and improved the image quality in coronary CTA. Benz et al.^60^ found that DLR improved image quality over HIR in coronary CT angiography but did not enhance diagnostic accuracy for stenosis compared to invasive angiography. Bernard et al.^59^ reported a 40% dose reduction with DLR compared to IR in cardiac CT angiography (CTDI_vol: 6.9 mGy vs 11.5 mGy), increasing SNR and CNR by 50%. The results of our study are in line with the above-mentioned studies and make a significant contribution to the narrow field of knowledge on the application of AI in noise reduction in CT imaging of the vascular system.

**Figure 6:**
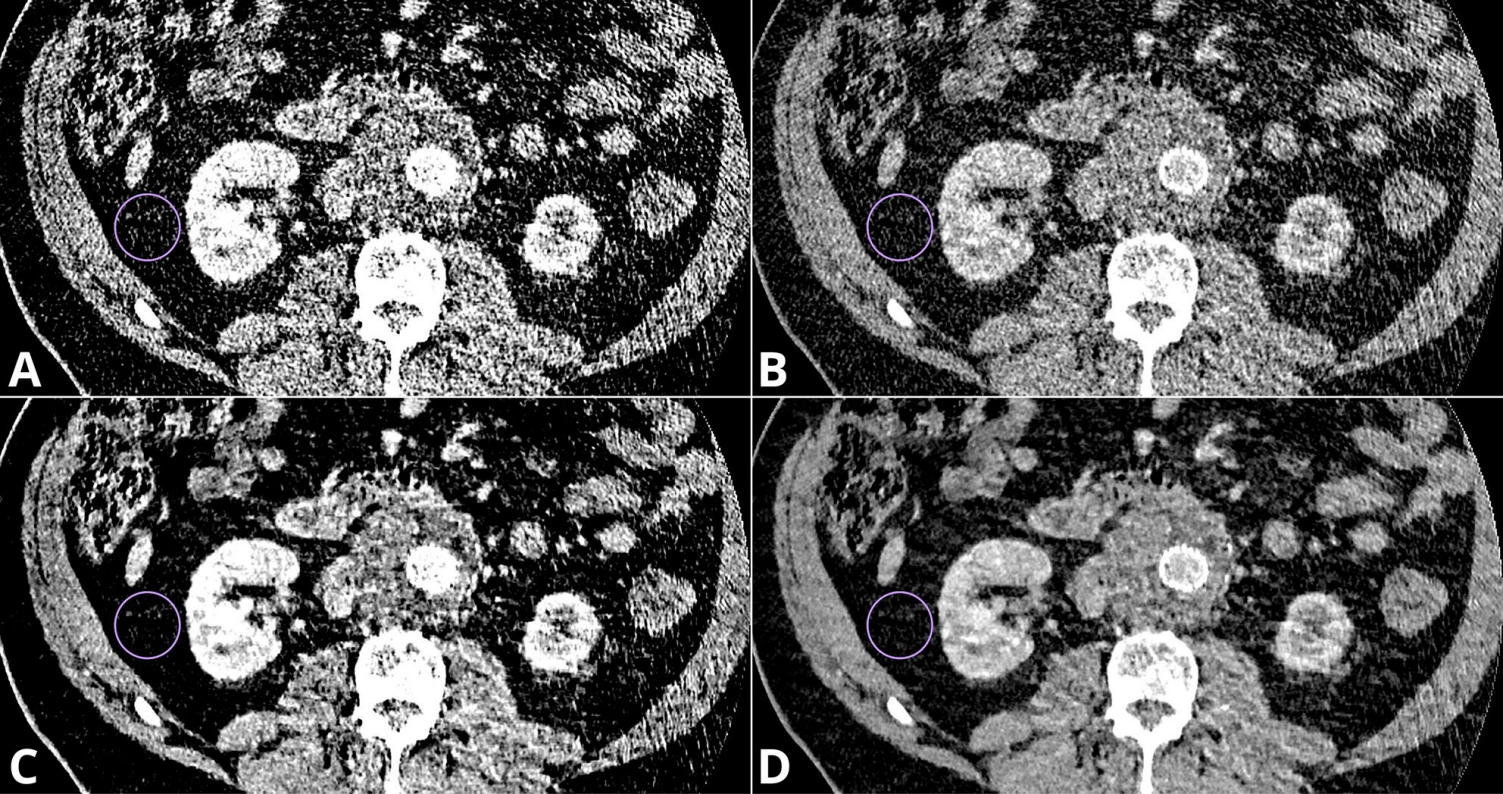
Sample of extremally high image noise level in reconstructions: VMI 40 keV – SD 105.5 (**A**); VMI 60 keV – SD 52.6 (**B**); DLM VMI 40 keV – SD 66.1 (**C**); DLM VMI 60 keV – SD 32.6 (**D**).

Figure 6 shows a case of extremely high image noise in an obese male patient (BMI = 33.1). Despite significant differences in image noise measurements, all six evaluated reconstructions were qualitatively rated as barely diagnostic; all six were unanimously marked by the readers with two points. This example clearly demonstrates that, despite improvements in objective image quality parameters, the overall subjective image quality might remain unchanged. The 2023 study on the use of metal artifact reduction (MAR) and IR reconstructions for stent visualization in f/bEVAR patients showed that, despite the high objective image quality parameters in MAR reconstructions, the subjective image quality was the worst in these reconstructions^50^. Both studies suggest that while the contrast-to-noise ratio (CNR) has undeniable value in the qualitative assessment of images, it is not the sole indicator determining the diagnostic value of the study. CNR primarily assesses contrast resolution but overlooks other critical aspects such as sharpness and spatial resolution, therefore other are crucial in comprehensive image quality assessment^61^.

The results of this study should be considered within the context of its limitations. First, the patient population was relatively small, yet sufficient for the analyses performed. Second, the results are specific to the DECT acquisition and postprocessing techniques, which are unique to one vendor. An IR with a specified potency was also employed, which affected the results of the image quality assessment. The subjective nature of image quality assessment can be influenced by individual biases. Finally, we only evaluated the DLM-based algorithm. Therefore, the results of this study should be regarded with an awareness of the specific hardware and software solutions applied.

## Conclusion

In conclusion, the use of DLM VMI images caused a significant increase in the diagnostic value of the examination due to a substantial increase in both subjective and objective image quality parameters. The use of iterative algorithms and DLM images increased the quantitative image parameters compared with VMI reconstructions and should be considered for inclusion in diagnostic protocols.

## Materials and methods

The study was approved by the Ethics Committee of Collegium Medicum, Nicolaus Copernicus University in Torun, Poland. The study was conducted in accordance with the Declaration of Helsinki and relevant guidelines. All patients provided written informed consent.

### Population

The study involved 28 consecutive patients who underwent the f/brEVAR procedure and were referred for 28 CTAs performed between August 2019 and December 2020. A follow-up examination was conducted for every patient 1 month after stentgraft implantation procedure. The exclusion criteria were: known severe adverse reactions to iodinated contrast media and impaired renal function (glomerular filtration rate < 30 mL/min).

### CT scanning protocol and image reconstruction

All CT scans were obtained using a dual-energy fast-kVp switching scanner (Discovery 750 HD, GE Healthcare, Milwaukee, WI, USA). The standard examination protocol consisted of three phases: one non-enhanced phase and two post-contrast dual-energy acquisitions (arterial and 60 s delayed-phase. Both post-contrast phases were acquired using the following tube parameters: tube voltage 80–140 kV, tube current 360 mAs, pitch 0.985:1, slice thickness 0.625 mm and a 35 cm DFOV. Intravenous administration of 80 mL of iohexol (350 mg I/ml), a non-ionic iodine contrast agent, was performed at a rate of 4 mL/min through the peripheral vein at the forearm. The contrast agent was followed by saline bolus chaser. A bolus tracking tool was used to trigger the start of arterial acquisition once the region of interest (ROI) in the proximal descending aorta exceeded 125 HU.

The delayed-phase axial images were reconstructed with VMI 40 and 60 keV images. Further, noise-optimized reconstructions were obtained:

1. Dedicated vendor-specific iterative reconstructions (ASIR; GE Healthcare) using a 60% strength level.
2. Vendor agnostic, DLM, software program (ClariCT.AI; ClariP, Seoul, South Korea)

Totally, 6 sets of reconstructions were obtained: 40, 60 keV VMI; IR 60% 40, 60 keV VMI; and DLM 40, 60 keV VMI.

All measurements were performed using a dedicated GE Healthcare console (GSI Viewer, Advantage Workstation Release 4.7, GE Healthcare).

### Assessment of endoleak presence and the subjective image quality

Subjective image quality assessment was conducted by three independent readers who were blinded to the type of reconstruction. These readers included board-certified specialists with 6 and 8 years of experience, and a radiology resident with 4 years of experience. The presence of totally 32 endoleaks was assessed prior to the reading sessions along with the slice levels at which they were best visualized. The readers were permitted to freely adjust the preset window settings. Readers were asked to rate the overall image quality of the reviewed reconstruction on a 5-point Likert scale:

1. Undiagnostic images,
2. Images of low diagnostic quality,
3. Images of acceptable diagnostic quality,
4. Images of good diagnostic quality,
5. Images of excellent diagnostic quality.

The presence of endoleaks was assessed on a five-point confidence scale:

1. Certainty that no endoleak was present,
2. Probable absence of an endoleak,
3. Presumed presence of an endoleak,
4. Probable presence of an endoleak,
5. Certain presence of an endoleak.

Intra-reader agreement regarding the results of subjective image quality was assessed.

### Assessment of objective image quality

Circular regions of interest (ROIs) were placed in the aortic lumen at the level of the main stent–graft module, visceral adipose tissue, one of the psoas muscles, and previously defined by consensus endoleaks. An automatic ROI propagation tool was used, “cloning” ROIs, in all of the investigated reconstructions. All measurements were performed by scaling the ROI as large as possible without calcifications, plaques, artifacts and stentgraft material. The mean attenuation and image noise, defined as the standard deviation (SD) in the subcutaneous adipose tissue, were registered. The contrast-to-noise ratio (CNR) and signal-to-noise ratio (SNR) were calculated as follows:

$$ {\mathbf{CNR}} = ({\text{A}}_{{{\text{A}},{\text{E}}}} {-}{\text{A}}_{{\text{p}}} )/{\text{N}} $$

$$ {\mathbf{SNR}} = {\text{A}}_{{{\text{A}},{\text{E}}}} /{\text{N}} $$

where A_A,E_ is the mean attenuation of the aortic lumen or endoleak, A_p_ is the mean attenuation of the psoas muscle, and N is noise (SD in the visceral adipose tissue).

### Error study

Ten randomly selected subjects were re-examined by the first author 1 month after the initial analysis. The ICC for subjective image quality analysis was calculated to assess the agreement between the two examinations.

### Statistical evaluation

The mean, standard deviation, median, quartiles, and range of quantitative variables were calculated. Paired Wilcoxon test was used to compare two repeated measures of quantitative variables. The inter-rater reliability of quantitative measures between readers was assessed using ICC 2 (according to Shrout and Fleiss). The significance level was set to 0.05. All analyses were conducted using the R software version 4.3.2.

## Data Availability

The data associated with our study are confidential due to the nature of medical records and imaging studies; however, access might be granted in justified cases. To gain access please contact the corresponding author.

## Abbreviations

AAA: Abdominal aortic aneurysms
EVAR: Endovascular aortic repair
f/bEVAR: Fenestrated/branched endovascular aortic repair
CTA: Computed tomography angiography
DECTA: Dual-energy computed tomography angiography
SECT: Single energy CT
VMI: Virtual monoenergetic image
keV: Kiloelectron volts
IR: Iterative reconstructions
ASIR: Adaptive statistical iterative reconstruction
FBP: Filtered back projection
DLR: Deep-learning-based image reconstruction algorithms
DLM: Deep learning model
GSI: Gemstone spectral imaging
ROI: Region of interest
HU: Hounsfield unit
CNR: Contrast-to-noise ratio
SNR: Signal-to-noise ratio
SD: Standard deviation
ICC: Intraclass correlation coefficient
MBIR: Model-based iterative reconstruction
MAR: Metal artifact reduction
BMI: Body mass index
CS: Confidence scale
